# Plasma microRNA signature associated with retinopathy in patients with type 2 diabetes

**DOI:** 10.1038/s41598-021-83047-w

**Published:** 2021-02-18

**Authors:** Donato Santovito, Lisa Toto, Velia De Nardis, Pamela Marcantonio, Rossella D’Aloisio, Alessandra Mastropasqua, Domenico De Cesare, Marco Bucci, Camilla Paganelli, Lucia Natarelli, Christian Weber, Agostino Consoli, Rodolfo Mastropasqua, Francesco Cipollone

**Affiliations:** 1 Department of Medicine and Aging Science, “G. d’Annunzio” University, Chieti, Italy; 2 Clinical Research Center, Center for Advanced Studies and Technologies (CAST), “G. d’Annunzio” University, Chieti, Italy; 3 Clinica Medica e Centro di Eccellenza Europeo e di Riferimento Regionale per l’Aterosclerosi, l’Ipertensione Arteriosa e le Dislipidemie, Nuovo Policlinico “SS. Annunziata”, Università “G. d’Annunzio” of Chieti, Via dei Vestini, 66, 66100 Chieti (CH), Italy; 4 Institute for Genetic and Biomedical Research, UoS of Milan, National Research Council, Milan, Italy; 5 German Centre for Cardiovascular Research (DZHK), Partner Site Munich Heart Alliance, Munich, Germany; 6 Institute for Cardiovascular Prevention (IPEK), Ludwig-Maximilians University, Munich, Germany; 7 Ophthalmology Clinic, “G. d’Annunzio” University, Chieti, Italy; 8 Munich Cluster for Systems Neurology (Synergy), Munich, Germany; 9 Department of Biochemistry, Cardiovascular Research Institute Maastricht (CARIM), University of Maastricht, Maastricht, The Netherlands; 10 Institute of Ophthalmology, University of Modena and Reggio Emilia, Via del Pozzo 71, 41124 Modena (MO), Italy

**Keywords:** Diabetes complications, Diabetes complications, Diagnostic markers

## Abstract

Diabetic retinopathy (DR) is a leading cause of vision loss and disability. Effective management of DR depends on prompt treatment and would benefit from biomarkers for screening and pre-symptomatic detection of retinopathy in diabetic patients. MicroRNAs (miRNAs) are post-transcriptional regulators of gene expression which are released in the bloodstream and may serve as biomarkers. Little is known on circulating miRNAs in patients with type 2 diabetes (T2DM) and DR. Here we show that DR is associated with higher circulating miR-25-3p (P = 0.004) and miR-320b (P = 0.011) and lower levels of miR-495-3p (P < 0.001) in a cohort of patients with T2DM with DR (n = 20), compared with diabetic subjects without DR (n = 10) and healthy individuals (n = 10). These associations persisted significant after adjustment for age, gender, and HbA1c. The circulating levels of these miRNAs correlated with severity of the disease and their concomitant evaluation showed high accuracy for identifying DR (AUROC = 0.93; P < 0.001). Gene ontology analysis of validated targets revealed enrichment in pathways such as regulation of metabolic process (P = 1.5 × 10^–20^), of cell response to stress (P = 1.9 × 10^–14^), and development of blood vessels (P = 2.7 × 10^–14^). Pending external validation, we anticipate that these miRNAs may serve as putative disease biomarkers and highlight novel molecular targets for improving care of patients with diabetic retinopathy.

## Introduction

Diabetic retinopathy (DR) is a microvascular complication of diabetes and a leading cause of vision disability and loss in the general population worldwide. It is caused by abnormal retinal blood vessels that are either proliferative (proliferative diabetic retinopathy, PDR) or functionally incompetent, leaking fluid and lipid into the retina. Visual impairment may occur when edema affects the central retina or macula (diabetic macular edema, DME) or even precede it, as in the case of vitreous hemorrhage or tractional retinal detachment. The prevalence of DR is approximately 30% of diabetic patients with an individual lifetime risk of DR around 60% in patients with type 2 diabetes (T2DM) and 90% in those with T1DM^1^. The prevalence of DME is higher in T2DM and is the primary cause of visual loss. Clinical trials demonstrated that vision loss as a consequence of severe DR can be minimized, in example, by intraocular anti-VEGF drugs for DME, laser photocoagulation for PDR. The effective treatment modalities available for early-stage disease shed a light on the relevance of the screening for DR in patients with diabetes. Hence, the identification of sensible and operator-independent DR biomarkers could improve detection of DR at earlier stages thus improving prognosis.

MicroRNAs (miRNAs) are short sequences (~ 22 nucleotides) of endogenous non-coding RNAs that emerged as a class of negative post-transcriptional modulators of gene expression. They act by guiding Argonaute proteins to the 3′ untranslated region (3′UTR) of target RNA messenger in the RNA-induced silencing complex (RISC), where multiple components (e.g. TNRC6, PABPC) contribute to post-transcriptional gene regulation by inhibiting translation or favoring RNA decay^2^. By fine-tuning the expression of multiple key biological targets, miRNAs play crucial roles in cell biology and their dysregulation has been associated with human diseases ranging from cancer to inflammatory, metabolic diseases, and cardiovascular diseases^3–5^. Noteworthy, miRNAs are also released in the extracellular space and detectable in biological fluids such as serum, plasma, urine, tears, aqueous and vitreous humors^6^. Experimental studies in vitro and in vivo as well as human studies provided evidence for a possible paracrine/endocrine function of circulating miRNAs (especially those included in exosomes)^6–11^. Moreover, the remarkable miRNA stability in biological fluids raised the intriguing possibility that they could serve as disease biomarkers.

Multiple studies have shown diabetes to influence circulating miRNAs expression^8,12,13^ and a role for miRNAs in DR could be deduced by preclinical studies^6^. More recently, the expression of 29 angiogenic miRNAs in patients with T1DM and DR in subgroups of the PREVENT-1 and PROTECT-1 cohorts has been reported^14^. However, although some investigation on circulating miRNAs in subject with T2DM and DR (*vs.* diabetic control individuals)^15–19^, little evidence exists on circulating miRNAs possibly associated with DR and its clinical severity also compared with non-diabetic individuals. Here, we report the results of a case–control study investigating circulating miRNAs in patients with different severity of DR aiming at finding a non-invasive diagnostic and prognostic “miRNA signature” of DR.

## Results

### Screening for differentially expressed circulating miRNAs

**Figure 1:**
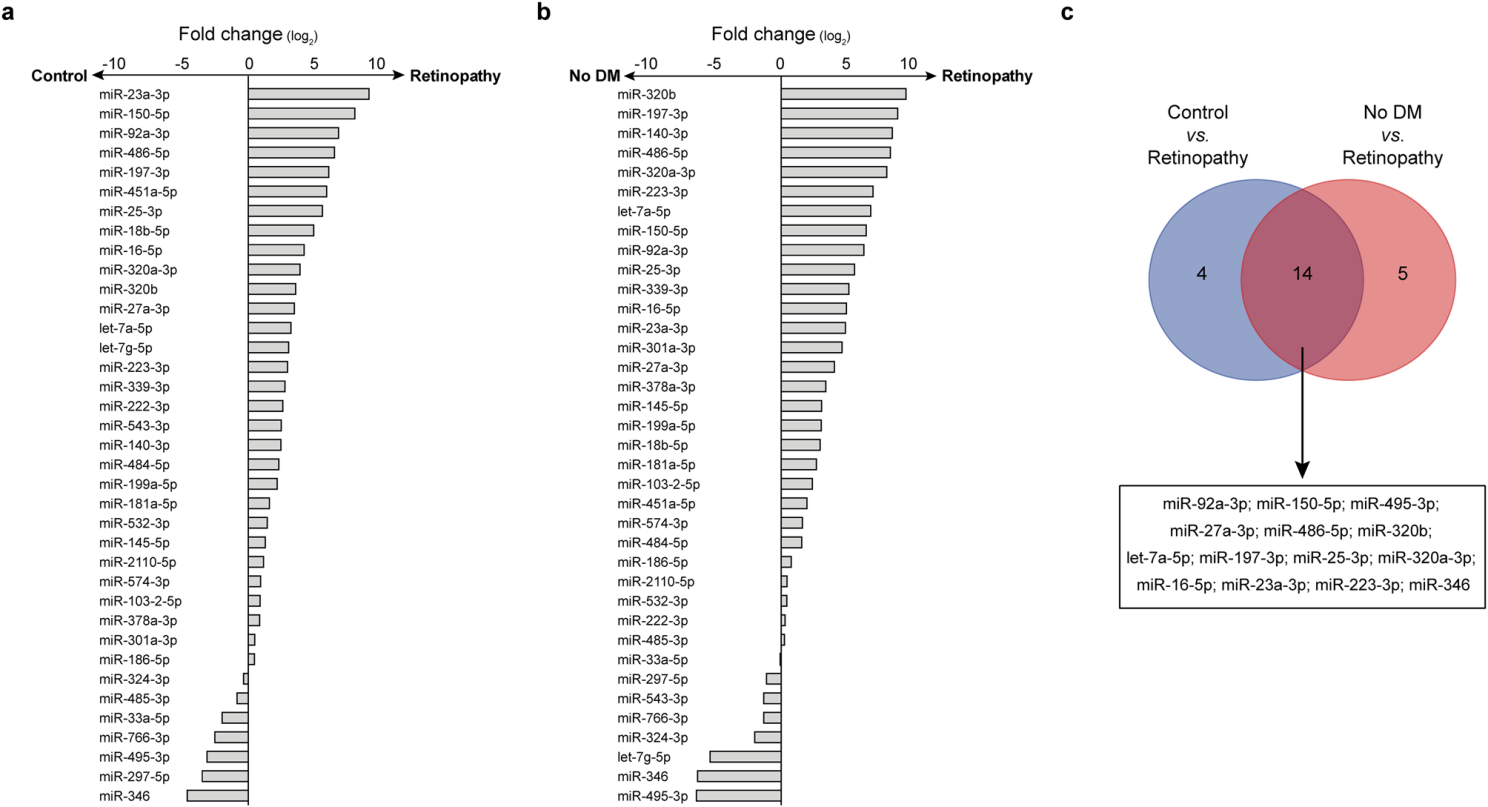
Circulating miRNome screening of DR. (**a**,**b**) Analysis of circulating miRNAs in pooled samples of diabetic patients with DR (Retinopathy) vs. diabetic patients without DR (Control) (**a**) or individuals without diabetes and retinopathy (No DM) (**b**). The x-axis indicates the fold changes on a log_2_ scale. DM, diabetes mellitus. (**c**) Venn’s diagram depicts the proportion of miRNAs consistently dysregulated in analyses in (**a**) and (**b**). The list of miRNAs is reported below.

For an initial screening, the expression of over 170 plasma-enriched miRNAs was assessed on samples obtained by pooling RNAs extracted from plasma exosomes of diabetic individuals without DR (Control) and with DR (Retinopathy). Type 2 diabetes is associated with profound changes in circulating (and exosomal) miRNAs^8,12,13^, hence an additional control group including individuals without diabetes and retinopathy was included in order to assess differences in miRNAs expression compared to the general population as well. Overall, 22.3% of the tested miRNAs were detected in all the pooled samples and we identified 18 circulating miRNAs potentially dysregulated (FC ≥ |8.0|) in patients with DR compared with diabetic individuals (Fig. 1a). Conversely, comparison of patients with DR with non-diabetic individuals showed dysregulation of 19 miRNAs (Fig. 1b). Among them, 12 miRNAs (let-7a-5p, miR-16-5p, miR-23a-3p, miR-25a-3p, miR-27a-3p, miR-92a-3p, miR-150-5p, miR-197-3p, miR-223-3p, miR-320a-3p, miR-320b, miR-486-5p) were upregulated and 2 miRNAs (miR-346 and miR-495-3p) showed a consistent downregulation in both comparisons (Fig. 1c). These 14 miRNAs and were selected for the following validation step.

### Internal validation of differential regulation

**Figure 2:**
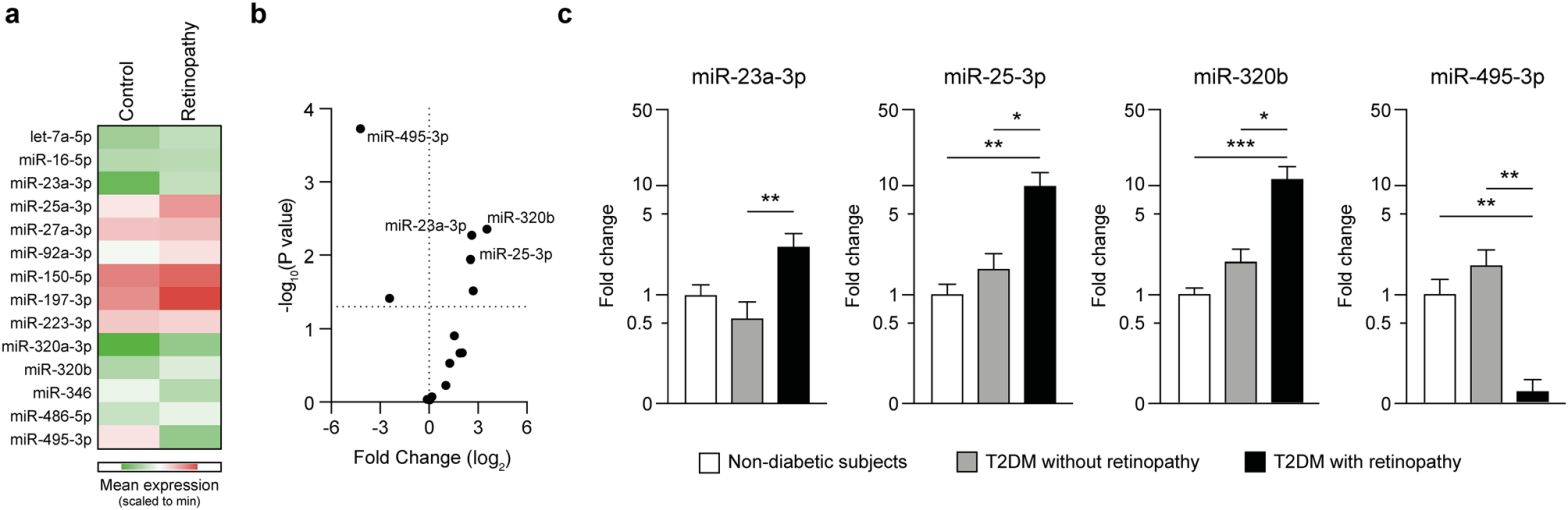
Validation of miRNA regulation by qPCR. (**a**,**b**) Expression of the selected miRNAs was performed by qPCR on diabetic patients without DR (Controls, n = 10) and with DR (Retinopathy, n = 20). Heatmap shows the mean expression of the miRNA in each group (**a**) and the Volcano plot reports the P-values by multiple *t*-tests (**b**). (**c**) Expression of the miRNAs showing a significant dysregulation in (**b**) measured in non-diabetic subjects (n = 10), diabetic patients without (n = 10) and with DR (n = 20). T2DM, type 2 diabetes. *p < 0.05; **p < 0.01; ***p < 0.001 as computed by univariate ANOVA with Bonferroni post-hoc test.

As analyses of pooled samples could not provide definitive results in terms of statistical significance, the expression of the selected miRNAs was measured in diabetic patients with and without DR. For this analysis, additional 10 diabetic patients with DR were included to enhance statistical power. Among the tested miRNAs, we found a significant upregulation of miR-23a-3p (log_2_FC = 2.65 ± 0.92, P = 0.005), miR-25-3p (log_2_FC = 3.54 ± 1.13, P = 0.004), and miR-320b (log_2_FC = 2.53 ± 0.93, P = 0.011), while miR-495-3p was dramatically downregulated (log_2_FC = − 4.21 ± 0.97, P < 0.001) (Fig. 2a,b). Of note, these differences remained significant also after accounting for multiple comparisons according to Benjamini–Hochberg FDR, while other miRNAs (miR-92a-3p and miR-346) were not confirmed as significant (Table S1). The expression of these 4 miRNAs was then compared to non-diabetic individuals: while expression of miR-25-3p (log_2_FC = 2.75 ± 0.99, P = 0.004), miR-320b (log_2_FC = 2.65 ± 0.54, P < 0.001), and miR-495-3p (log_2_FC = -3.25 ± 0.89, P = 0.003) were significantly different, we did not detect significant differences in miR-23a-3p expression (log_2_FC = 1.83 ± 0.91, P = n.s.) between patients with DR and non-diabetic subjects (Fig. 2c).

### Association of miRNAs with severity of DR

**Figure 3:**
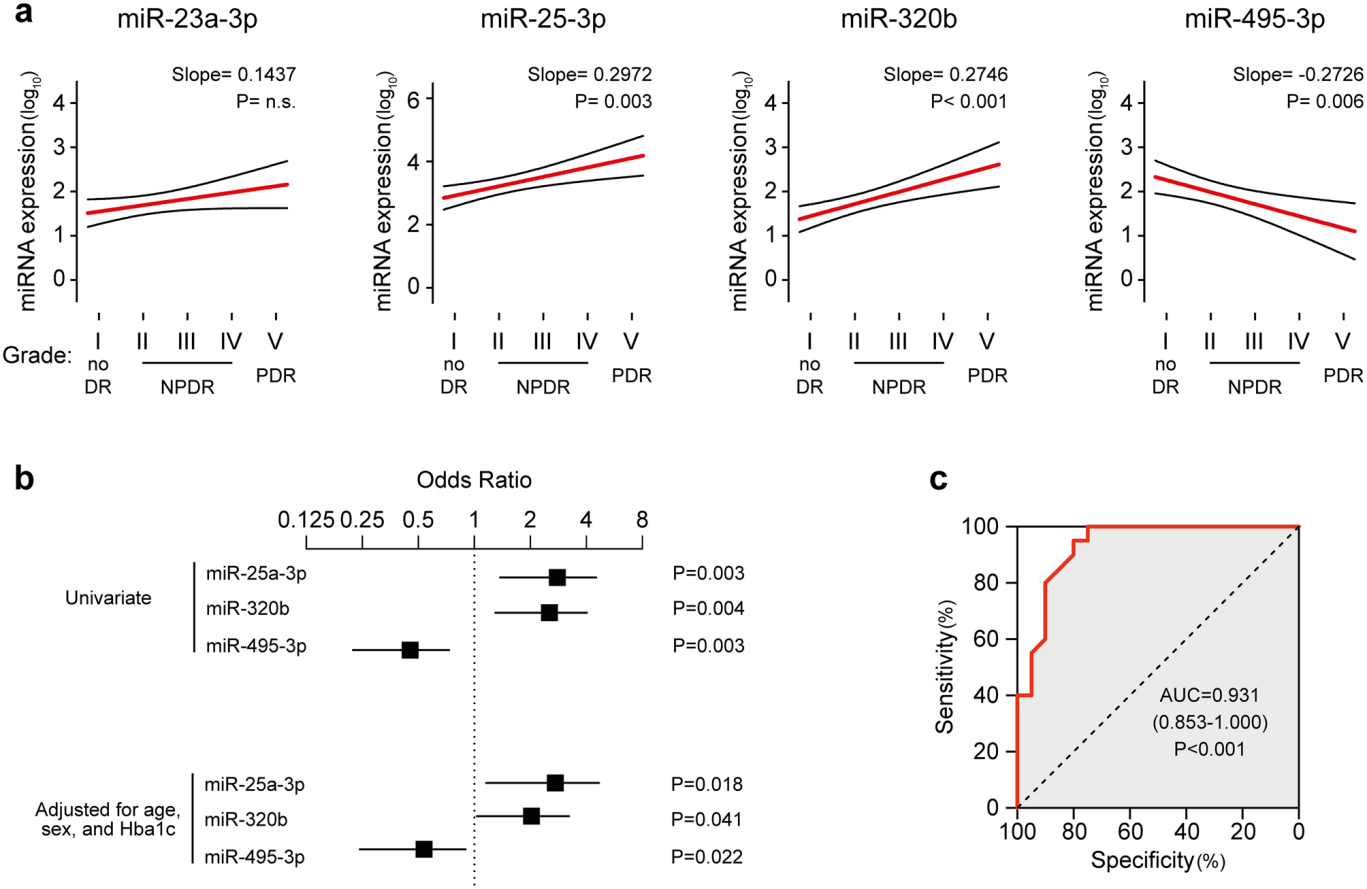
Circulating miR-25-3p, miR-320b and miR-495-3p are associated with DR. (**a**) Univariate linear regression models showing log-linear trends of association between miRNAs and severity of DR. Grade I, no retinopathy (n = 10); Grade II, mild no-proliferative DR (NPDR, n = 5); Grade III, moderate NPDR (n = 5); Grade IV, severe NPDR (n = 7); Grade V, proliferative DR (n = 3). (**b**) Forest plot showing odds ratios and the 95% CI for association of the single miRNAs with presence of DR in univariate logistic models (upper) or after correction for age, sex, and HbA1c (lower). (**c**), Receiver-operating characteristic (ROC) curve derived from the logistic regression model developed on circulating miR-25-3p, miR-320b, and miR-495-3p. AUC, area under the curve.

Next, we investigate the association between circulating miRNAs expression and DR severity, staged according to the International Clinical Disease Severity Scale for DR. Interestingly, significant moderate grade correlations between severity of DR and circulating miR-25-3p (Spearman’s ρ = 0.52, P = 0.001), miR-320b (ρ = 0.51, P = 0.001), and miR-495-3p (ρ = − 0.46, P = 0.003) were found, showing a linear trend in regression analyses (Fig. 3a) which was independent on age and gender (Table S2). Conversely, no significant correlation was observed for miR-23a-3p (ρ = 0.28, P = n.s.). Moreover, logistic regression analyses confirmed the significant associations of circulating miR-25-3p (Odds ratio, OR = 2.50, 95% confidence interval = 1.38–4.52, P = 0.003), miR-320b (OR = 2.29, 1.30–4.03, P = 0.004), and miR-495-3p (OR = 0.40, 0.22–0.73, P = 0.003) with DR, that persisted after adjustment for age, gender, and HbA1c in multivariate logistic regression models (Fig. 3b, Table S3). Furthermore, no significant correlations were detected between circulating miR-23a-3p, miR-25-3p, miR-320b, miR-495-3p, and age, nor these miRNAs displayed sex-specific regulation (Fig. S1, Table S4). Finally, since combination of multiple miRNAs could yield superior discriminative performance than individual miRNAs, we conducted logistic regression analysis employing the circulating level of miR-25-3p, miR-320b, and miR-495-3p as independent variables. The overall model resulted statistically significant (Cox and Snell R^2^ = 0.526, P < 0.001) and the derived Receiver Operating Characteristic (ROC) curve demonstrated a good accuracy of this model for statistically detecting DR in our study cohort (AUC = 0.931, 0.853–1.000, sensitivity and specificity 85%, P < 0.001) (Fig. 3c) and correlated with DR severity with larger coefficients (ρ = 0.79, P < 0.001) than individual miRNAs.

Lastly, we explored the association between DME, a relevant cause of vision loss that could occur at any stage of DR^1^, and dysregulated miRNAs. Logistic regression analyses revealed significant associations for miR-320b (OR = 2.41, 1.27–4.56, P = 0.007) and miR-495-3p (OR = 0.46, 0.23–0.94, P = 0.035). Entering both miRNAs as covariates yielded a statistically significant model (Cox and Snell R^2^ = 0.287, P = 0.001) with a good diagnostic performance as revealed by ROC curve analysis (AUC = 0.847, 0.722–0.972, sensitivity 83%, specificity 79%, P < 0.001).

### Prediction of biological relevance

**Figure 4:**
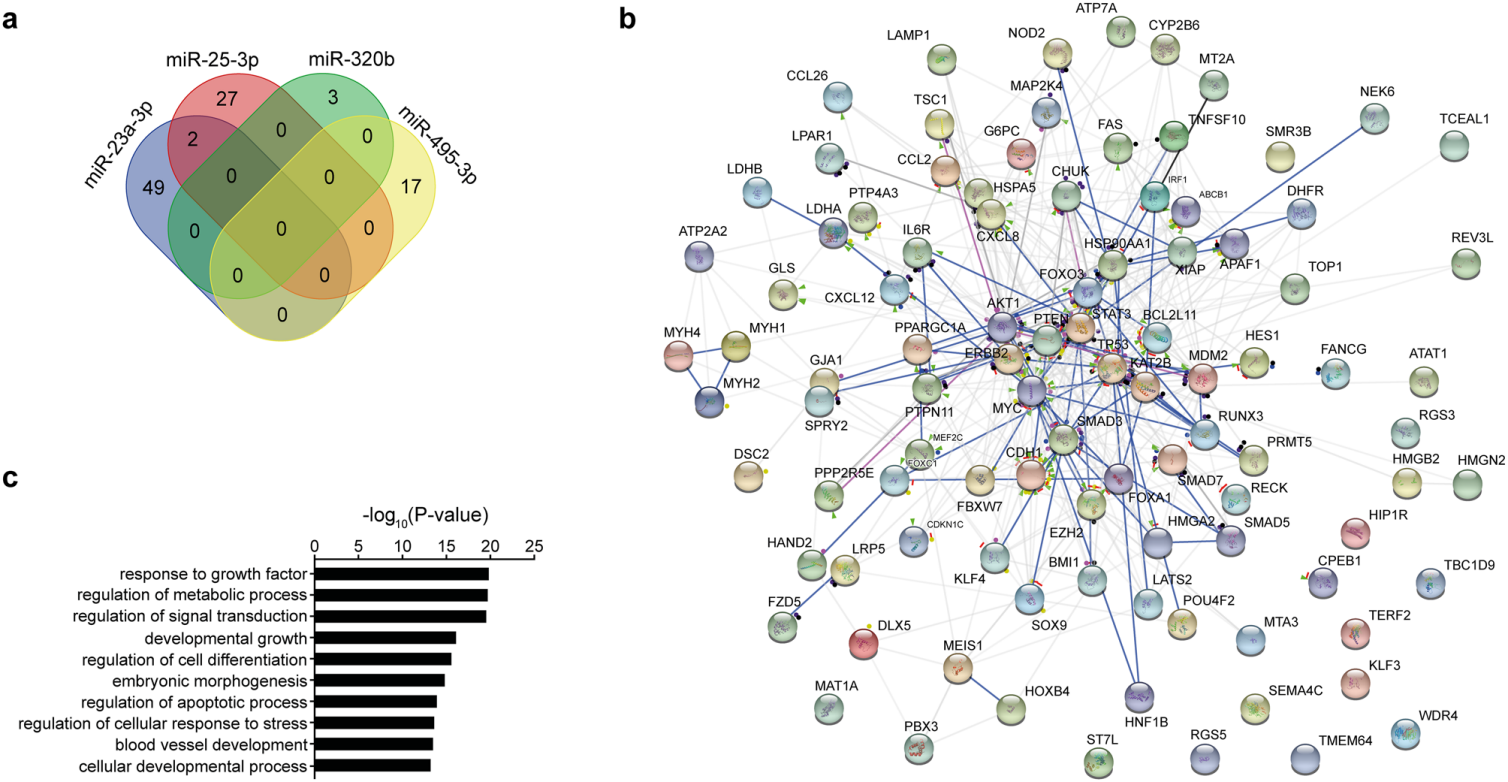
Biological pathway network. (**a**) Venn’s diagram for the overlap of target mRNA for the dysregulated miRNAs. (**b**) Network analysis of biochemical interaction among the validated targets of the dysregulated miRNAs as enlisted in the STRING database. (**c**) Gene ontology analysis for enrichment in biological processes pathways of the validated targets of the dysregulated miRNAs.

Circulating miRNAs in exosomes have been proposed as a paracrine (and potentially endocrine) intercellular communication system^6–10^. Hence, we explored the network of the biochemical interactions involving the validated targets of the dysregulated miRNAs. The list of miRNA targets with functional validation is reported in Table S5. Intriguingly, a low target overlap was found as only miR-23a-3p and miR-25-3p shared two common targets (CDH1 and PTEN) (Fig. 4a). The complete network of biochemical interactions is shown in Fig. 4b. Of note, gene ontology analysis revealed these targets to be involved in crucial pathways including regulation of metabolic process, regulation of cell response to stress, and development of blood vessels (Fig. 4c). Moreover, mapping the targets to the REACTOME database identified a significant enrichment in processes relevant for endothelial cell proliferation^20^, such as the regulation of the NOTCH1 (*e.g.* R-HSA-4641262, Fold enrichment = 11.5, P = 0.027) and the β-catenin pathways (e.g. R-HSA-2559580, Fold enrichment = 4.9, P = 0.018). Together, these data suggest a possible biological relevance of the dysregulated miRNAs in influencing (endothelial) cell proliferation and—possibly—angiogenesis.

## Discussion

With the increasing prevalence of T2DM, the worldwide relevance of DR as a major cause of vision loss demands efforts for early diagnosis and treatment. In this study, we provide preliminary evidence that patients with T2DM and DR exhibit peculiar changes in circulating miRNAs conveyed by exosomes. In particular, we found higher expression of miR-25-3p and miR-320b, and lower levels of miR-495-3p compared with non-diabetic individuals and patients with T2DM without DR. Interestingly, the expression of these miRNAs correlated with the severity of the disease. These associations were not dependent on age, sex and glycemic control (reflected by circulating HbA1c) and the concomitant evaluation of the 3 miRNAs provided good accuracy for categorizing patients affected by DR in our study cohort.

Few studies have previously investigated the expression of miRNAs in diabetic patients with DR. A large nested case–control study explored the expression of 29 circulating miRNAs in two prospective cohorts of patients with T1DM (PROTECT-1 and PREVENT-1). This study exploited the associations of miR-27b-3p and miR-320a-3p with the incidence of DR^14^. In our study, another member of the miR-320 family (namely, miR-320b) was significantly upregulated, whereas changes in miR-320a-3p did not reach statistical significance. Notably, while miR-27b-3p and miR-320a-3p were highly expressed in endothelial cells and may affect angiogenesis^14^, cell ontology in the FANTOM5 database suggests that the miRNAs identified in our study are enriched in other cell types (mainly fibroblasts, stem cells and leukocytes)^21^. Hence, distinct pathways and miRNAs may underlie the development of DR in the context T1DM or T2DM. Future experimental studies will be needed to properly investigate these mechanisms.

Circulating miRNAs in whole plasma and serum of patients with T2DM and DR has also been investigated. Several studies specifically evaluate a single miRNA, while some performed wide expression profiling by RNA-sequencing mainly including diabetic patients as reference group^22^. In particular, Liang et al.^18^ performed a 3-step validation study to identify higher circulating let-7a-5p, miR-28-3p and miR-novel-chr5-15976 in patients with early-stage DR. Noteworthy, a tendential (although not significant) increase of let-7a-5p was also observed in our cohort (which included DR with different grade of severity). Nonetheless, we and others have previously reported lower circulating let-7a-5p in patients with T2DM^8,12^. The opposite regulation in patients with T2DM vs. non-diabetic subjects and in diabetic patients with or without DR could limit the use of let7a-5p as a biomarker for DR. Future studies on larger prospective T2DM cohorts will help to identify the optimal miRNA signature for diagnosis of DR.

In our study we specifically investigated circulating miRNAs carried by vesicles, including exosomes. Exosomes are particularly interesting in biology as their biogenesis involves convoluted intracellular protein complexes to produce a selective loading of miRNAs and other cargoes^11^. Previous studies suggested that exosomes could effectively deliver miRNAs into recipient cells to affect biological response in a paracrine/endocrine communication system^6–11^. To our knowledge, only Mazzeo et al.^23^ investigated exosome-carried miRNAs in patients with DR revealing the dysregulation of miR-150-5p, miR-21-3p and miR-30b-5p and clearly proved in vitro the functional relevance in crucial pathophysiological features of DR. Although Mazzeo et al. investigated patients affected by T1DM undergoing insulin treatment^23^, their results support the existence of a regulatory pathway exerted by vesicular miRNAs in DR. Remarkably, the validated targets for the miRNAs identified in our study are implicated in important pathways for pathogenesis of DR, including regulation of response to growth factors, metabolic processes, cell differentiation, response to stress and blood vessels development. Hence, it is possible that the dysregulated miRNAs reflect an active underlying intracellular communication leading (or compensating) the development of DR.

Some limitations of our study should be addressed by following investigations. Although encouraging, our findings derive from a small cohort, thus the evaluation of these miRNAs in independent, multicentric and prospective studies will be required to definitively validate the diagnostic performance of these miRNAs as biomarker of DR before progressing into clinical practice to possibly modify management protocols. Moreover, functional studies (ideally in vivo) should verify their ultimate biological relevance in processes underlying the development of DR. Finally, some patients enrolled in the study underwent intravitreal injections prior to the study. Although preliminary post-hoc analyses on our data do not show a clear influence of intravitreal injection, whether treatments for DR could influence or reverse the observed changes in circulating miRNAs should be addressed in appropriate interventional clinical trials. Nevertheless, we believe that our findings are potentially important because they point out the existence of a peculiar circulating miRNA profile in patients with DR with possible pathophysiological relevance which could be exploited as a tool for diagnostic screening and follow-up. These aspects may ultimately lead to a better management of DR by facilitating screening and allow early identification of patients that would benefit of treatment.

## Methods

### Study population

In this prospective case–control study, we enrolled 30 T2DM patients classified according to the Clinical Diabetic Retinopathy Scale proposed by the Diabetic Retinopathy Project Group^24^ as diabetic patients without retinopathy (n = 10) and with DR (n = 10) referring to the University “G. d’Annunzio”, Chieti-Pescara, Italy. Ten non-diabetic subjects were enrolled as an additional control group. The validation cohort has been further expanded with the inclusion of additional 10 diabetic patients with DR to increase the statistical power for analyses on different severity grades of disease. We excluded patients with history or clinical evidence of active inflammatory diseases, cancer, liver/kidney dysfunction. Moreover, ocular exclusion criteria were (a) intravitreal injections of anti-vascular endothelial growth factor (VEGF) or steroids in either eye in the previous 9 months; (b) inflammatory eye diseases; (c) diagnosis of glaucoma; (d) ocular surgery and retinal laser treatment in the previous 9 months; (e) significant media opacities (cataract, vitreous hemorrhage). Clinical characteristics of the study cohort are depicted in Table 1. The study was approved by Ethics Committee of the “G. d’Annunzio” University of Chieti-Pescara. All participants provided their written informed consent, and all procedures were carried out in accordance with the ethics standards of the institutional and/or national research committee and with the 2013 Helsinki Declaration and its later amendments.

**Table 1:** Study population.

	Controls (n = 10)	Diabetic patients	
		Without retinopathy (n = 10)	With retinopathy (n = 20)
**General**			
Age (years)	55.3 ± 10.6*	69.6 ± 8.7	66.3 ± 8.3
HbA_1_c			
(%)	5.8 ± 0.3*	6.9 ± 0.4	7.1 ± 0.8
(mmol/mol)	40.0 ± 3.3*	52.0 ± 4.4	54.0 ± 8.7
Systolic blood pressure (mmHg)	134.7 ± 15.8	130.0 ± 3.2	134.5 ± 13.3
Diastolic blood pressure (mmHg)	83.2 ± 11.0	84.2 ± 4.9	83.3 ± 8.9
Sex (M/F)	6/4	5/5	10/10
Hypertension (n)	0^$^	3	5
Hypercholesterolemia (n)	2	2	1
**Ocular**			
Best corrected visual acuity (LogMAR)	− 0.03 ± 0.05	− 0.02 ± 0.04	0.37 ± 0.19*
Diabetic macular edema (n)	0	0	12
Previous retinal laser surgery (n)	0	0	12
Previous intravitreal injection (n)	0	0	16

Continuous variables are reported as mean ± standard deviation; *p < 0.01 for comparison with other groups by ANOVA with Bonferroni post-hoc test. ^$^p < 0.05 for comparison with other groups by Pearson χ^2^. No other statistically significant differences revealed.

### Clinical assessment of diabetic retinopathy

All patients underwent a comprehensive ophthalmic evaluation, including assessment of best corrected visual acuity (BCVA), tonometry, slit-lamp biomicroscopy, and indirect fundus ophthalmoscopy. Diabetic retinopathy stage was defined according to the Clinical Diabetic Retinopathy Scale proposed by the Diabetic Retinopathy Project Group using retinal digital photographs^24^. Stages are as follows: (I) no DR; (II) mild non proliferative diabetic retinopathy (NPDR); (III) moderate NPDR; (IV) severe NPDR; (V) PDR.

### Blood sampling, exosome isolation and RNA extraction

Whole blood was drawn into EDTA tubes and plasma was separated by centrifugation. Plasma exosomes were precipitated using the ExoQuick exosome precipitation solution (System Biosciences) from 250 µL of plasma samples, then RNA was extracted employing a miRNeasy kit (Qiagen), as previously described^8,25^.

### Quantitative polymerase chain reaction (qPCR)

Quantification of miRNA expression was carried out as previously described^8,25–27^. A preliminary miRNome screening analysis was performed employing a Serum/Plasma Focus miRNA PCR panel (Exiqon) on three homogenous samples obtained by pooling a fixed volume (2 µL) of RNA extracted from the exosomes of (a) type 2 diabetic patients without clinical evidence of retinopathy (n = 10), (b) type 2 diabetic patients with confirmed diabetic retinopathy (n = 10), (c) control subjects without diabetes and retinal diseases (n = 10). After plate calibration with the internal spike-in, data were normalized to the global mean of Cq values of miRNA. miRNA with fold changes (FC) ≥ |8.0| between groups were measured on individual RNA samples. For internal validation, a fixed volume of 4 µL of RNA from each subject was reverse transcribed into cDNA by means of a miRCURY LNA™ cDNA synthesis kit (Exiqon) and expression of the miRNAs was assessed by qPCR using miRCURY LNA™ miRNA PCR assays. miR-19-5p and miR-125a-5p were identified as the most stable references miRNAs by employing the geNorm and NormFinder algorithms^28,29^ and used for normalization of expression data.

### Bioinformatics

Validated miRNA-target interactions were obtained consulting miRTarBase v.8 (Ref.^30^). Non-functionally tested as well as functionally weak interactions were excluded. Gene ontology and pathway enrichment were analyzed by using multiple platforms, including the Database for Annotation, Visualization and Integrated Discovery (DAVID) v. 6.8 (Ref.^31^), Metascape (Ref.^32^), and the REACTOME database v.71 (Ref.^33^). The representation of the interaction network was achieved by including the biochemical interactions enlisted in the STRING database v.11 (Ref.^34^).

### Statistical analysis

Statistics were calculated using SPSS 25 (IBM Corp.) and Prism 9 (Graphpad Inc.). Log-transformed values were used for all computations. After transformation, data distribution and homogeneity of variance were tested by the Shapiro–Wilk and Levene’s test, respectively. Comparisons between 2 groups were performed by Student’s *t* test, with Welch correction when appropriate. The probability values were corrected for multiple comparisons by using Benjamini–Hochberg approach with a false discovery rate (FDR) set as 5%. Univariate ANOVA with Bonferroni post-hoc test was applied to assess significance in comparisons among three or more groups. Differences in proportions were assessed by Pearson χ^2^ test. Correlations were estimated by Spearman correlation test and linear regression. Logistic regression analyses, aimed at identifying the ability of deregulated miRNAs to correctly classify the study subjects, were performed as described^8,25,35^. For circulating miRNAs, quintiles of expression were included for computing odds ratios. ROC curve analysis was carried out in order to verify the goodness of model fitting on our study population. A two-tailed P-value < 0.05 was deemed as statistically significant. Unless otherwise noted, descriptive statistics included mean and standard error from mean (s.e.m.) or 95% Confidence Intervals (95% CI). The statistical test is reported in the respective figure legends.

## Supplementary Information

Supplementary Information.

## Data Availability

The data that support the findings of this study are presented in the current manuscript and in its figures. Data are available from the corresponding authors, upon reasonable request.
