# Supplementary material for: Plasma microRNA signature associated with retinopathy in patients with type 2 diabetes
Source: Sci Rep. 2021 Feb 18;11:4136. doi: 10.1038/s41598-021-83047-w (PMC7892881; doi:10.1038/s41598-021-83047-w)
Supplement: Supplementary file 1 — Supplementary Information. [file 41598_2021_83047_MOESM1_ESM.pdf]

Supplementary Material for

**Plasma microRNA signature associated with retinopathy in patients with type 2 diabetes**

Donato Santovito*, Lisa Toto*, Velia De Nardis*, Pamela Marcantonio, Rossella D'Aloisio, Alessandra Mastropasqua, Domenico De Cesare, Marco Bucci, Camilla Paganelli, Lucia Natarelli, Christian Weber, Agostino Consoli, Rodolfo Mastropasqua[§] and Francesco Cipollone[§]

**Supplementary Table S1.** Differentially expressed circulating miRNAs in patients with diabetic retinopathy

| *microRNA* | *$log_2FC$* | *SE of difference* | *P value* | *q value* | *Significant after FDR?* |
|---|---|---|---|---|---|
| *miR-23a-3p* | 2.65 | 0.92 | 0.0052 | 0.0243 | Yes |
| *miR-25-3p* | 3.54 | 1.13 | 0.0043 | 0.0243 | Yes |
| *miR-92a-3p* | 2.71 | 1.18 | 0.0297 | 0.0833 | No |
| *miR-320b* | 2.53 | 0.93 | 0.0112 | 0.0392 | Yes |
| *miR-346* | -2.43 | 1.11 | 0.0378 | 0.0884 | No |
| *miR-495-3p* | -4.21 | 0.97 | 0.0001 | 0.0025 | Yes |

Comparisons were assessed by multiple Student's t-test and probability values were corrected for multiple comparisons by using Benjamini-Hochberg approach with a false discovery rate (FDR) set as 5%. Comparisons yielding a q-value < 0.05 were deemed as statistically significant after FDR.

**Supplementary Table S2.** Summary of multivariate linear regression models

**(A) miR-25a-3p**

| ***Variables in the model*** | ***Beta-coefficient*** | ***P value*** |
|---|---|---|
| *Circulating miR-25a-3p* | 0.48 | 0.0032* |
| *Age* | 0.14 | 0.3544 (n.s.) |
| *Sex* | 0.41 | 0.4147 (n.s.) |

**(B) miR-320b**

| ***Variables in the model*** | ***Beta-coefficient*** | ***P value*** |
|---|---|---|
| *Circulating miR-320b* | 0.52 | 0.0011* |
| *Age* | 0.07 | 0.6348 (n.s.) |
| *Sex* | 0.08 | 0.5727 (n.s.) |

**(C) miR-495-3p**

| ***Variables in the model*** | ***Beta-coefficient*** | ***P value*** |
|---|---|---|
| *Circulating miR-495-3p* | -0.43 | 0.0064* |
| *Age* | 0.16 | 0.2782 (n.s.) |
| *Sex* | 0.04 | 0.8175 (n.s.) |

Dependent variable: severity of diabetic retinopathy (DR): grade I, no DR; grade II, mild not-proliferative DR; Grade III, moderate NPDR; Grade IV, severe NPDR; Grade V, proliferative DR. Circulating miRNAs expression are log-transformed. Simultaneous-entry of covariates was employed.

**Supplementary Table S3.** Summary of multivariate logistic regression models

**(A) miR-25a-3p**

| ***Variables in the model*** | ***Odds Ratio*** | ***95% CI*** | ***P value*** |
|---|---|---|---|
| *Circulating miR-25a-3p* | 2.37 | 1.16 – 4.85 | 0.0179* |
| *Age* | 0.91 | 0.80 – 1.05 | 0.1797 (n.s.) |
| *HbA1c* | 2.70 | 0.36 – 20.30 | 0.3336 (n.s.) |
| *Sex* | 2.71 | 0.34 – 21.52 | 0.3446 (n.s.) |

**(B) miR-320b**

| ***Variables in the model*** | ***Odds Ratio*** | ***95% CI*** | ***P value*** |
|---|---|---|---|
| *Circulating miR-320b* | 1.95 | 1.03 – 3.70 | 0.0411* |
| *Age* | 0.92 | 0.82 – 1.04 | 0.1644 (n.s.) |
| *HbA1c* | 2.45 | 0.34 – 17.85 | 0.3738 (n.s.) |
| *Sex* | 1.65 | 0.26 – 10.31 | 0.5943 (n.s.) |

**(C) miR-495-3p**

| ***Variables in the model*** | ***Odds Ratio*** | ***95% CI*** | ***P value*** |
|---|---|---|---|
| *Circulating miR-495-3p* | 0.46 | 0.23 – 0.89 | 0.0220* |
| *Age* | 0.97 | 0.87 – 1.09 | 0.6164 (n.s.) |
| *HbA1c* | 2.21 | 0.52 – 9.40 | 0.2849 (n.s.) |
| *Sex* | 2.00 | 0.00 – 13.34 | 0.4731 (n.s.) |

**Supplementary Table S4.** Association between circulating miRNAs and age

| ***microRNA*** | ***Spearman's Rho*** | ***P value*** | ***Significant?*** |
|---|---|---|---|
| *miR-23a-3p* | - 0.052 | 0.748 | No |
| *miR-25-3p* | + 0.147 | 0.366 | No |
| *miR-320b* | + 0.239 | 0.138 | No |
| *miR-495-3p* | - 0.021 | 0.896 | No |

Bivariate correlation's coefficients were estimated by Spearman's test.

**Supplementary table S5.** Functionally validated microRNAs' targets

| miRNA | Target | Experimental evidence |
|---|---|---|
| **miR-23a-3p** | CXCL12 | Luciferase reporter assay; Reporter assay; Other |
| | HES1 | Immunofluorescence; Luciferase reporter assay; Northern blot; Western blot |
| | POU4F2 | Luciferase and GFP reporter assay; Northern blot; qPCR; Western blot |
| | ATAT1 | Luciferase reporter assay |
| | IL6R | GFP reporter assay; Microarray; qPCR; Western blot |
| | PPARGC1A | Luciferase reporter assay |
| | G6PC | Luciferase reporter assay |
| | FOXO3 | Immunoblot; Luciferase reporter assay; qPCR |
| | FANCG | Immunoblot; Luciferase reporter assay; qPCR; Western blot |
| | MYH1 | Luciferase reporter assay; qPCR; Western blot |
| | MYH2 | Luciferase reporter assay; qPCR; Western blot |
| | MYH4 | Luciferase reporter assay; qPCR; Western blot |
| | PTEN | Luciferase reporter assay; qPCR; Western blot; Flow cytometry |
| | PTEN | Luciferase reporter assay |
| | PTPN11 | Luciferase reporter assay |
| | HMGN2 | Luciferase reporter assay |
| | LDHA | Luciferase reporter assay; qPCR |
| | CDH1 | qPCR; Western blot |
| | KLF3 | Luciferase reporter assay; Western blot |
| | IRF1 | Luciferase and GFP reporter assay; qPCR; Western blot; Immunofluorescence |
| | FAS | Flow cytometry; Luciferase reporter assay; Microarray; Western blot |
| | MT2A | Immunofluorescence; Immunohistochemistry; In situ hybridization; Luciferase reporter assay; qPCR; Western blot |
| | PPP2R5E | GFP reporter assay; qPCR; Western blot |
| | LDHB | Luciferase reporter assay; qPCR |
| | CXCL8 | Luciferase reporter assay; qPCR; Western blot |
| | APAF1 | Luciferase reporter assay; qPCR; Western blot; Flow cytometry |
| | NEK6 | Luciferase reporter assay |
| | LPAR1 | Luciferase reporter assay; qPCR; Western blot |
| | FZD5 | qPCR; Microarray; Western blot |
| | TOP1 | Luciferase reporter assay; qPCR; Western blot; Flow cytometry |
| | HOXB4 | Luciferase reporter assay; Western blot |
| | LAMP1 | Luciferase reporter assay; qRT-PCR |
| | HMGB2 | Luciferase reporter assay; qPCR; Western blot |
| | SPRY2 | Luciferase reporter assay; qPCR; Western blot; ChIP-seq |
| | MEF2C | Luciferase reporter assay |
| | HIP1R | Luciferase reporter assay |
| | XIAP | Luciferase reporter assay; qPCR; Western blot |

| | | |
|---|---|---|
| | LRP5 | Luciferase reporter assay; qPCR; Western blot |
| | TMEM64 | Luciferase reporter assay; Western blot |
| | HSP90AA1 | Luciferase reporter assay; qPCR; Immunofluorescence |
| | HNF1B | qRT-PCR//Microarray//Western blot |
| | TSC1 | Luciferase reporter assay//qRT-PCR//Western blot//Microarray |
| | SMAD3 | Luciferase reporter assay; qPCR; Western blot; Immunofluorescence |
| | STAT3 | Luciferase reporter assay; Immunofluorescence; Western blot |
| | GJA1 | Illumina Expression Arrays; Luciferase reporter assay; qPCR; Western blot |
| | FOXA1 | Luciferase reporter assay; qPCR; Western blot |
| | SMAD5 | Luciferase reporter assay; qPCR; Western blot |
| | CHUK | Luciferase reporter assay |
| | ST7L | Luciferase reporter assay |
| | TERF2 | Luciferase reporter assay |
| | RGS5 | Luciferase reporter assay |
| | GLS | Luciferase reporter assay; qPCR; Western blot |
| **miR-25-3p** | PRMT5 | Western blot |
| | BCL2L11 | Microarray; Luciferase reporter assay; qPCR; Western blot |
| | KLF4 | Luciferase reporter assay; Western blot |
| | CDKN1C | Luciferase reporter assay; qPCR; Western blot |
| | KAT2B | Luciferase reporter assay; Western blot |
| | TP53 | Luciferase reporter assay; qPCR; Western blot |
| | WDR4 | Luciferase reporter assay; qPCR; Western blot |
| | CDH1 | Luciferase reporter assay; qPCR; Western blot |
| | CCL26 | Luciferase reporter assay; qPCR; Western blot |
| | MDM2 | Luciferase reporter assay; qPCR; Western blot |
| | PTEN | Luciferase reporter assay |
| | EZH2 | Luciferase reporter assay; qPCR; Western blot |
| | SMAD7 | Luciferase reporter assay; qPCR; Western blot |
| | MAP2K4 | Western blot |
| | DSC2 | Luciferase reporter assay; Western blot |
| | ATP2A2 | In situ hybridization; Luciferase reporter assay; qPCR; Western blot |
| | RECK | Luciferase reporter assay; qPCR; Western blot |
| | TCEAL1 | Luciferase reporter assay; qPCR; Western blot; Flow cytometry |
| | LATS2 | Luciferase reporter assay; qPCR; Western blot |
| | REV3L | Luciferase reporter assay |
| | FBXW7 | Luciferase reporter assay; qPCR; Western blot; Immunohistochemistry |
| | HAND2 | Luciferase reporter assay |
| | CPEB1 | Luciferase reporter assay |
| | TNFSF10 | Western blot |
| | CYP2B6 | Luciferase reporter assay; qPCR; Western blot; EMSA |

| | | |
|---|---|---|
| | RGS3 | Luciferase reporter assay; qPCR; Western blot |
| | DHFR | Luciferase reporter assay; qPCR; Western blot |
| | SEMA4C | Luciferase reporter assay; qPCR; Western blot |
| **miR-320b** | MYC | Luciferase reporter assay; qPCR; Western blot |
| | DLX5 | Luciferase reporter assay; qPCR; Western blot |
| | NOD2 | Luciferase reporter assay; qPCR; Western blot |
| **miR-495-3p** | PBX3 | Luciferase reporter assay; qPCR; Western blot; Microarray; Immunohistochemistry |
| | MEIS1 | Luciferase reporter assay; qPCR; Western blot; Microarray; Immunohistochemistry |
| | MTA3 | Luciferase reporter assay; qPCR; Western blot; |
| | SOX9 | Luciferase reporter assay; Microarray; qPCR; Western blot; safranin O staining/GAGs contents assay |
| | PTP4A3 | qPCR; Western blot |
| | BMI1 | Luciferase reporter assay; qPCR; Western blot; Microarray; Immunohistochemistry |
| | SMR3B | qPCR; Western blot |
| | ATP7A | GFP reporter assay; qPCR; Western blot |
| | TBC1D9 | Luciferase reporter assay; qPCR |
| | MAT1A | Luciferase reporter assay |
| | HSPA5 | Luciferase reporter assay; qPCR |
| | FOXC1 | GFP reporter assay; qPCR; Western blot |
| | CCL2 | ELISA; qPCR; Luciferase reporter assay; Western blot |
| | RUNX3 | LacZ reporter assay; Microarray; qPCR; Western blot; Luciferase reporter assay |
| | AKT1 | Luciferase reporter assay; qPCR; Western blot |
| | HMGA2 | Luciferase reporter assay; qPCR; Western blot |
| | ABCB1 | Luciferase reporter assay; qPCR; Western blot |

**Supplementary Figure S1.** Circulating miRNAs expression per sex category

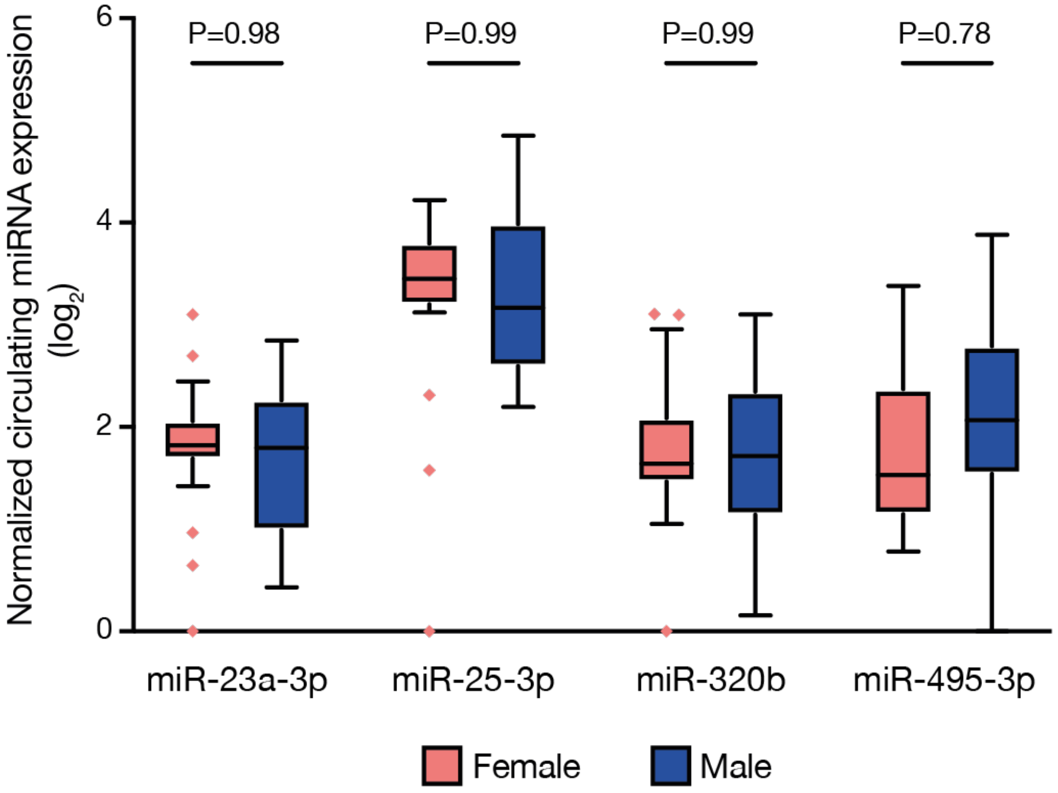


Expression of the circulating miRNAs showing a significant dysregulation in patients with diabetic retinopathy was evaluated by qPCR. Data have been stratified by sex and P-values were computed by multiple *t*-tests. No statistically significant differences were observed.
